# A Randomized Pilot Trial of Remote Ischemic Preconditioning in Heart Failure with Reduced Ejection Fraction

**DOI:** 10.1371/journal.pone.0105361

**Published:** 2014-09-02

**Authors:** Michael A. McDonald, Juarez R. Braga, Jing Li, Cedric Manlhiot, Heather J. Ross, Andrew N. Redington

**Affiliations:** 1 Division of Cardiology, Peter Munk Cardiac Centre, University of Toronto, Toronto, Canada; 2 Division of Cardiology, Hospital for Sick Children, University of Toronto, Toronto, Canada; Kurume University School of Medicine, Japan

## Abstract

**Background:**

Remote ischemic preconditioning (RIPC) induced by transient limb ischemia confers multi-organ protection and improves exercise performance in the setting of tissue hypoxia. We aimed to evaluate the effect of RIPC on exercise capacity in heart failure patients.

**Methods:**

We performed a randomized crossover trial of RIPC (4×5-minutes limb ischemia) compared to sham control in heart failure patients undergoing exercise testing. Patients were randomly allocated to either RIPC or sham prior to exercise, then crossed over and completed the alternate intervention with repeat testing. The primary outcome was peak VO2, RIPC versus sham. A mechanistic substudy was performed using dialysate from study patient blood samples obtained after sham and RIPC. This dialysate was used to test for a protective effect of RIPC in a mouse heart Langendorff model of infarction. Mouse heart infarct size with RIPC or sham dialysate exposure was also compared with historical control data.

**Results:**

Twenty patients completed the study. RIPC was not associated with improvements in peak VO2 (15.6+/−4.2 vs 15.3+/−4.6 mL/kg/min; p = 0.53, sham and RIPC, respectively). In our Langendorff sub-study, infarct size was similar between RIPC and sham dialysate groups from our study patients, but was smaller than expected compared to healthy controls (29.0%, 27.9% [sham, RIPC] vs 51.2% [controls]. We observed less preconditioning among the subgroup of patients with increased exercise performance following RIPC (p<0.04).

**Conclusion:**

In this pilot study of RIPC in heart failure patients, RIPC was not associated with improvements in exercise capacity overall. However, the degree of effect of RIPC may be inversely related to the degree of baseline preconditioning. These data provide the basis for a larger randomized trial to test the potential benefits of RIPC in patients with heart failure.

**Trial Registration:**

ClinicalTrials.gov +++++NCT01128790

## Introduction

Exercise impairment in patients with chronic heart failure (HF) is associated with significant morbidity and mortality [1]–[4]. Objective measures of exercise capacity continue to be important predictors of clinical outcomes, and are widely used to risk stratify HF patients for advanced therapies, including cardiac transplantation [3]–[5]. The effects of chronic left ventricular dysfunction and HF on exercise capacity are multifactorial, and relate to diminished cardiac output and to alterations in peripheral and respiratory skeletal muscle structure and function [6]–[9]. Notably, improvements in skeletal muscle function and exercise capacity are associated with improvements in left ventricular function [10], reduced hospitalization [11], and better transplant-free survival [12]. It follows that interventions to improve exercise capacity may translate into significant clinical benefits in the HF population.

Remote ischemic preconditioning (RIPC) is a well-described protective mechanism in which a transient, sub-lethal reduction in blood flow to tissues in one area of the body renders other remote tissues more resistant to subsequent episodes of prolonged ischemia [13]. Although the precise mechanisms are not fully defined, the remote ischemic preconditioning stimulus appears to trigger release of circulating factors [14]–[16] that result in downstream effects on mitochondrial function within the target organ(s) [13]. Early clinical application of RIPC in cardiac patients has been encouraging; transient limb ischemia, typically involving 2–4 cycles of 5–10 minutes each, has been shown to limit myocardial injury in the setting of elective cardiac or vascular surgery, as well as percutaneous coronary intervention for angina or acute myocardial infarction [17]–[21].

To date, the impact of RIPC in HF patients has not been explored. However, ‘local’ ischemic preconditioning of the legs improved maximal power output and maximal oxygen consumption during subsequent bicycle ergometry in healthy volunteers [22]. Recent work from members of our group has demonstrated that RIPC can improve maximal performance in highly trained swimmers, suggesting a salutary effect on peripheral skeletal, respiratory and cardiac muscle function under conditions of exercise induced hypoxic ischemia [23]. We therefore sought to determine whether RIPC would be associated with improvements in exercise capacity in patients with HF due to left ventricular systolic dysfunction.

## Methods

The protocol for this trial and supporting CONSORT checklist are available as supporting information; see Checklist S1 and Protocol S1.

### Ethics Statement

All patients provided written informed consent, and the study protocol was approved by the Research Ethics Board of the University Health Network, and registered prior to initiation (Identifier: NCT01128790, registered December 2009 at ClinicalTrials.gov). The CONSORT 2010 Statement for clinical trials was followed for the reporting of our study results herein. There are currently no ongoing clinical trials of RIPC intervention from our group at this time.

### Patient Population

We performed a randomized, controlled, crossover pilot trial of patients recruited from the Heart Function Clinic at the Toronto General Hospital, a University of Toronto affiliated teaching hospital. The Heart Function Clinic is a large volume multi-disciplinary clinic that manages of a broad spectrum of HF patients, including those with advanced HF referred for consideration of transplantation or mechanical circulatory support.

Ambulatory clinic patients scheduled for routine cardiopulmonary stress testing were approached for study inclusion if they met all of the following criteria: ≥18 years of age, left ventricular ejection fraction (LVEF) <40%, history of New York Heart Association (NYHA) class II–IV symptoms, HF for at least 12 weeks, and clinical stability. Patients were initially excluded for any of the following reasons: ischemic cardiomyopathy, cardiovascular related hospitalization within the preceding four weeks, diabetes, peripheral neuropathy, inability to exercise or other contraindications to stress testing, inability or unwillingness to participate in serial exercise testing. Patients were enrolled and followed from September 2009 to February 2011. Following study initiation, there were comparatively few patients from our clinic population with non-ischemic cardiomyopathy who met all other inclusion criteria, and we therefore modified our protocol to include patients with stable underlying coronary disease that were otherwise eligible. All other exclusion criteria were maintained throughout the study.

### Study Protocol

Patients were approached for participation and consent was obtained prior to their scheduled cardiopulmonary exercise test. Immediately prior to testing, patients were randomized by one of the investigators (MM or JB) in a 1∶1 ratio by computer generated allocation sequence, to the order in which they received either RIPC intervention or sham intervention. Patients then crossed over to receive the alternate intervention at a dedicated follow-up visit for repeat exercise testing. Where possible, patients were scheduled for a second exercise study within four weeks to minimize the potential impact of disease progression or any interim therapeutic intervention on exercise capacity. Throughout the duration of the trial, patients were unaware of the expected effects of the sham or RIPC treatment and investigators (other than those performing the intervention) remained blinded to treatment assignment.

The RIPC stimulus consisted of four cycles of 5 minutes of upper limb ischemia followed by 5 minutes of reperfusion. A blood pressure cuff was placed over the upper arm, and limb ischemia was achieved by inflating the cuff 20 mmHg above the systolic pressure. The sham intervention consisted of four cycles of 5 minutes of blood pressure cuff inflation to only 10 mmHg, interspersed with 5 minutes of cuff deflation to simulate reperfusion. After completing four cycles of either intervention, 30 mL of blood was drawn, placed on ice and transferred to the investigators' laboratory as part of the pre-specified mechanistic sub-study. Immediately thereafter, patients completed a cardiopulmonary exercise test with respiratory gas analysis according to a routine stationary bicycle ramp protocol (10 Watt per minute incremental workload). Testing was symptom limited after achieving a diagnostic workload, defined as a respiratory exchange ratio >1.05. A dedicated exercise physiologist who was unaware of the treatment assignment supervised all tests, and a blinded investigator (HR) reported the results.

The primary outcome measure was peak oxygen consumption (VO2) measured in milliliters of oxygen consumed per kilogram of body mass per minute (mL/kg/min) during peak exercise following sham intervention compared to peak VO2 following RIPC intervention. Secondary outcomes included a comparison of exercise duration, workload achieved, ventilatory anaerobic threshold, absolute peak VO2, and the slope of the minute ventilation (VE) to carbon dioxide production (VCO2) ratio. As this pilot study was exploratory in nature and undertaken for feasibility, no sample size calculations were performed. We estimated that 20 patients with paired exercise data would represent an adequate sample to inform future trial design and generate appropriate study hypotheses.

### Sub-study Protocol

We conducted a pre-specified sub-study to assess whether HF patients elaborate circulating protective factor(s) in response to a RIPC stimulus. The animal protocols used for this study were approved by the Animal care and Use Committee of the Hospital for Sick Children in Toronto and conformed with the Guide for the Care and Use of Laboratory Animals published by the National Institutes of Health (publication # 85-23, revised 1996). The 30 mL of blood drawn immediately following the RIPC or sham intervention was collected in heparinized tubes and placed on ice. It was subsequently centrifuged at 3000 rpm for 20 minutes at room temperature and the plasma fraction was dialyzed against a 20 fold volume of Krebs-Henseleit solution across a 12–14 kDa dialysis membrane. The dialysate was then prepared for use in a mouse heart Langendorff model as previously described [23]. Briefly, the dialysate was made isotonic and adjusted to pH 7.4 with sodium bicarbonate and glucose. Prior to perfusion of the mouse hearts, D-glucose, NaHCO3 and EDTA were added to a final concentration of 15 mM, 25 mM and 0.5 mM in the dialysate, respectively. Mice were anesthetized with pentobarbital in standard fashion and the hearts were excised, cannulated at the aorta, and perfused with modified Krebs-Ringer buffer at 37°C. A water-filled latex balloon was inserted into the left ventricle, connected to a pressure transducer and maintained at 7–10 mmHg to allow beat to beat measurement of left ventricular pressures (PowerLab data acquisition system AD Instruments; Colorado Springs, CO, USA). Dialysate derived from each patient for both sham and RIPC conditions was used to perfuse 2–4 mouse hearts, and data from each preparation was averaged.

Following a 20 minute stabilization period, the hearts were perfused with the study patient dialysate solution and then subjected to 30 minutes of no-flow ischemia followed by 60 minutes of reperfusion. After completing the protocol, the hearts were frozen at −80°C. Each heart was then sectioned into 1 mm thick slices and stained with 1.25% 2,3,5-triphynyltetrazolium chloride to distinguish areas of infarcted tissue (white colour) from non-infarcted tissue (red colour). The slices were formalin fixed and scanned into Photoshop. Using this method, it was possible to trace the area of infarcted tissue and express this as a proportion of the total area of LV myocardium at risk. The primary endpoint of this Langendorff sub-study was infarct area in hearts perfused with sham dialysate versus infarct area in hearts perfused with RIPC dialysate. Secondary endpoints included LV developed pressure, LV end-diastolic pressure, maximum rate of systolic pressure rise (dP/dt max) and fall (dP/dt min). Infarct size observed after sham or RIPC dialysate exposure in heart failure patients described above was also compared with infarct size observed using an identical sham and RIPC procedure to prepare dialysate from 4 healthy subjects serving as historical controls (4 males, age range 25–50 years).

### Statistical Analysis

Continuous data are reported as means with standard deviation, and categorical data are reported as frequencies. Normality of the distribution of continuous variables was tested using the Shapiro-Wilks test. Differences in exercise outcomes between sham control and RIPC intervention were assessed using paired t-tests. Logistic regression model was used to identify factors associated with response to RIPC, with relative difference between RIPC and Sham used as a dependent variable and each categorical variable or each tertile of continuous variables as independent predictors. A p-value of <0.05 was considered significant for all comparisons. All analyses were performed using SPSS for Windows v 11.0 (SPSS Inc., Chicago, IL).

## Results

### Main Study

**Figure 1:**
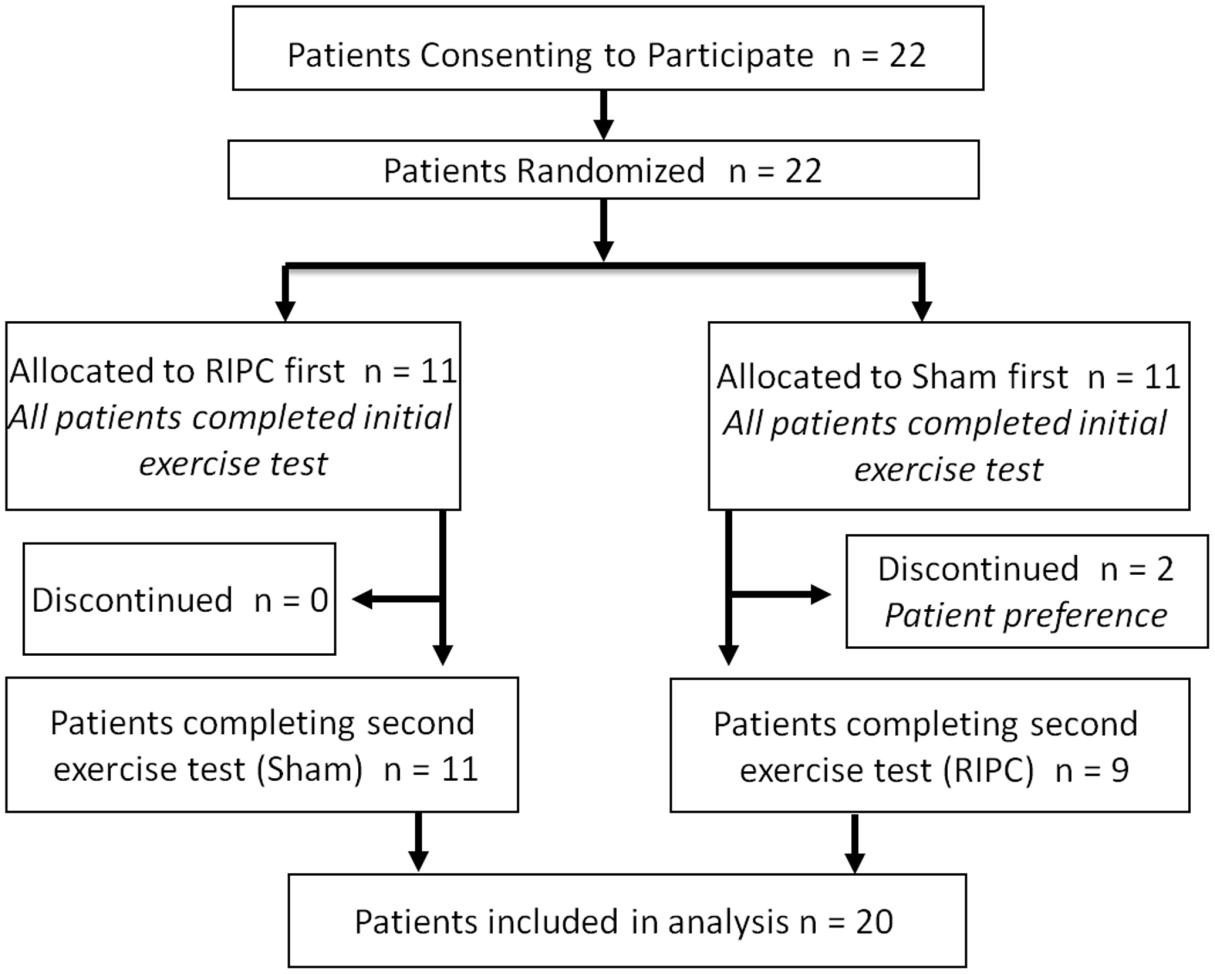
Patient flow through the study. 22 patients consented to participate in the study. Two patients declined repeat testing and were excluded from the analysis; 20 patients complete the study protocol with paired testing.

Figure 1 outlines the patient flow through the study. In total, 22 patients consented to participate from September 2009 to January 2011, and all 22 were randomized and subsequently completed the assigned first intervention prior to undergoing the initial cardiopulmonary exercise stress test. Two patients, both allocated to the initial sham control group, withdrew from the study and declined to undergo follow-up exercise testing. Therefore, the final study population included in the analysis consisted of 20 patients who completed the protocol with both sham and RIPC interventions.

The baseline characteristics of the study patients are shown in Table 1. As outlined, the majority were males with non-ischemic cardiomyopathy. All patients were treated with beta blockers and ACE inhibitors, 70% required loop diuretics, and a large proportion of patients had received a defibrillator and/or cardiac resynchronization therapy. Although all patients had previously been characterized as NYHA class II, III, or IV, most were minimally symptomatic at the time of enrollment.

**Table 1:** Baseline characteristics of the study population.

**Age** (years)	56.3+/−11.8
**Male**	18 (90)
**Duration of heart failure** (months)	71.3+/−63.4
**Etiology:**	
Ischemic	4 (20)
Non-ischemic	16 (80)
**LVEF** (%)	29.3+/−6.8
**NYHA functional class:**	
I	5 (25)
II	10 (50)
III	5 (25)
IV	-
**HTN** *	3 (15)
**Smoking history:**	
Never	11 (55)
Prior	8 (40)
Current	1 (5)
**Atrial fibrillation**	6 (30)
Medications:	
ACE inhibitor/ARB†	20 (100)
Beta blocker	20 (100)
Spironolactone	10 (50)
Digoxin	7 (35)
Loop diuretic	14 (70)
Nitrate	2 (10)
**AICD** ‡	12 (60)
**CRT** ‖	5 (25)
**Systolic blood pressure** (mmHg)	105.7+/−12.8
**Diastolic blood pressure** (mmHg)	69+/−9.9
**Heart rate** (beats/min)	64.8+/−7.8
**Height** (cm)	178.6+/−8.6
**Weight** (kg)	94.7+/−22.5
**Hemoglobin** (g/L)	145.4+/−14.8
**Serum creatinine** (µmol/L)	94+/−22.5
**Serum sodium** (mmol/L)	138.5+/−2.7
**BNP** # (pg/mL)	Range 10.0–1616.0

(N = 20).

Values are expressed as means +/− standard deviation, or numbers and percentages.

*HTN: hypertension.

† ARB: angiotensin receptor blocker.

‡ AICD: automated implantable cardioverter-defibrillator.

‖ CRT: cardiac resynchronization therapy.

# BNP: B-type natriuretic peptide.

**Figure 2:**
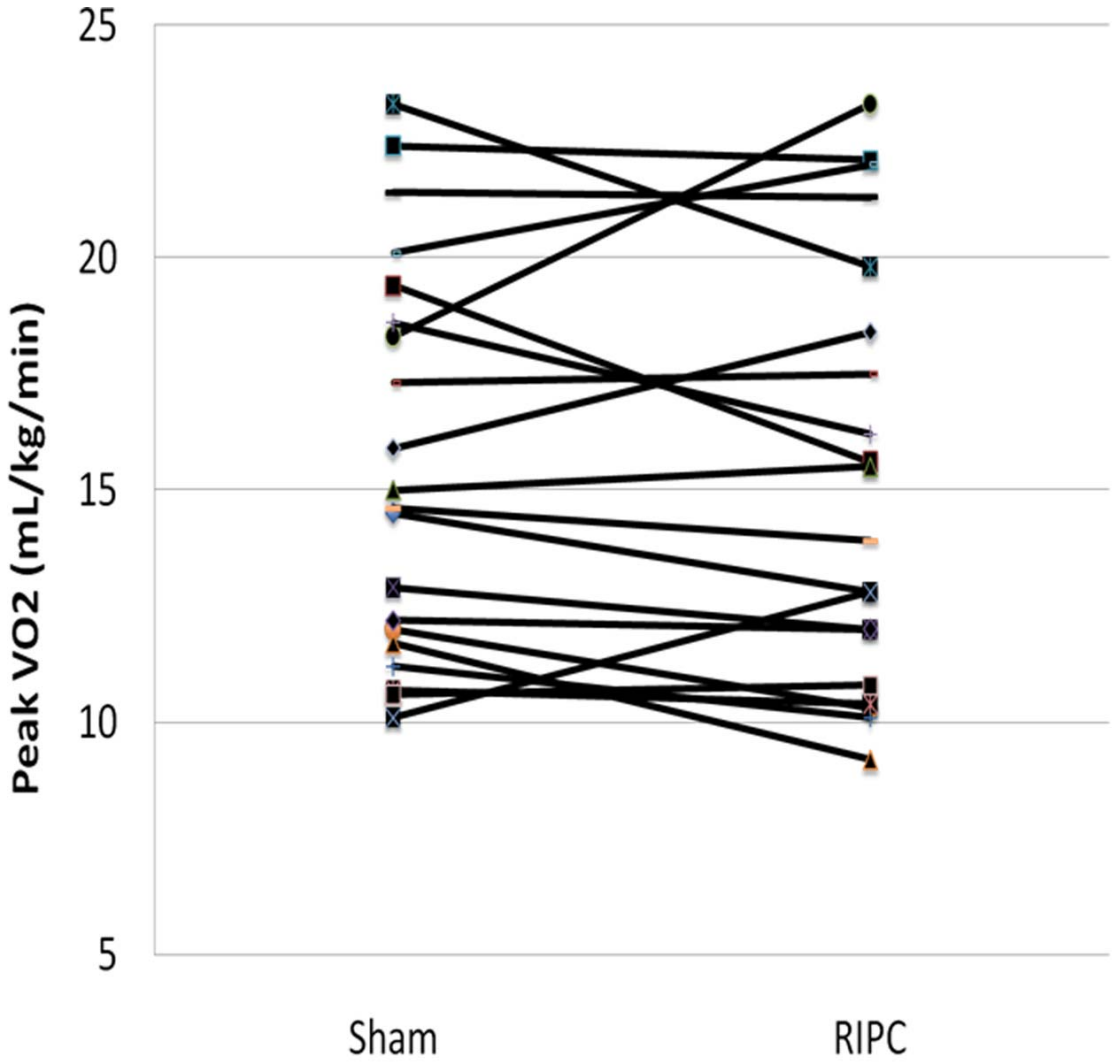
Individual exercise test results for sham versus RIPC intervention. Peak VO2 is shown for all study subjects undergoing exercise stress testing immediately following sham and RIPC interventions.

The majority of patients in this study (16/20) completed sequential cardiopulmonary exercise tests within 30 days as planned. Two patients were unable to return for scheduled follow-up testing for logistical reasons and completed the protocol within 90 days, and an additional two patients completed follow-up by 180 days. There were no adverse effects observed for any of our study patients. The results for the primary outcome, peak VO2 for RIPC compared to sham intervention, are shown in Figure 2 and Table 2. Overall, RIPC prior to cardiopulmonary exercise testing was not associated with improvements in peak VO2 in this population (15.6+/−4.2 mL/kg/min vs 15.3+/−4.6 mL/kg/min; p = 0.53, for sham and RIPC, respectively). With respect to the secondary outcomes, there was no observed benefit of RIPC on exercise duration, workload achieved, anaerobic threshold, or VE/CO2 slope (Table 2). Excluding patients that did not complete the protocol within 30 days had no impact on our results. Moreover, post-hoc exploratory analysis did not identify any baseline clinical characteristics, (including age, gender, duration of heart failure, functional class, BNP level) associated with an improved peak VO2 following RIPC intervention. Finally, the sequence of testing did not appear to have an effect on our results, as there were no significant differences in peak VO2 from the initial exercise study compared to the follow-up study, irrespective of the treatment allocation.

**Table 2:** Exercise performance of Sham control versus RIPC intervention.

	Sham	RIPC	P value
**Peak VO2** (ml/kg/min)	15.6+/−4.2	15.3+/−4.6	0.53
**Secondary Endpoints:**			
Exercise duration (minutes)	10.3+/−3.0	11.0+/−3.0	0.13
Workload (watts)	108.5+/−29.8	103.0+/−32.6	0.07
AT* (ml/kg/min)	9.6+/−2.6	9.6+/−3.0	0.98
Peak VO2† (L/min)	1.45+/−0.36	1.42+/−0.42	0.39
VE/VECO2‡ slope	28.7+/−4.3	29.9+/−5.4	0.06

(N = 20).

Values expressed as means +/− standard deviation.

*AT: anaerobic threshold.

† VO2: oxygen uptake.

‡ VE/VCO2: minute ventilation - carbon dioxide production ratio.

### Langendorff Sub Study

In the Langendorff study, there was no significant difference in infarct area between mouse hearts perfused with sham dialysate and those perfused with RIPC dialysate (29.0+/−7.9% versus 27.9+/−6.8%; p = 0.62). Additionally, the secondary endpoint hemodynamic measurements were not significantly different at any stage following perfusion with either sham or RIPC derived dialysate.

**Figure 3:**
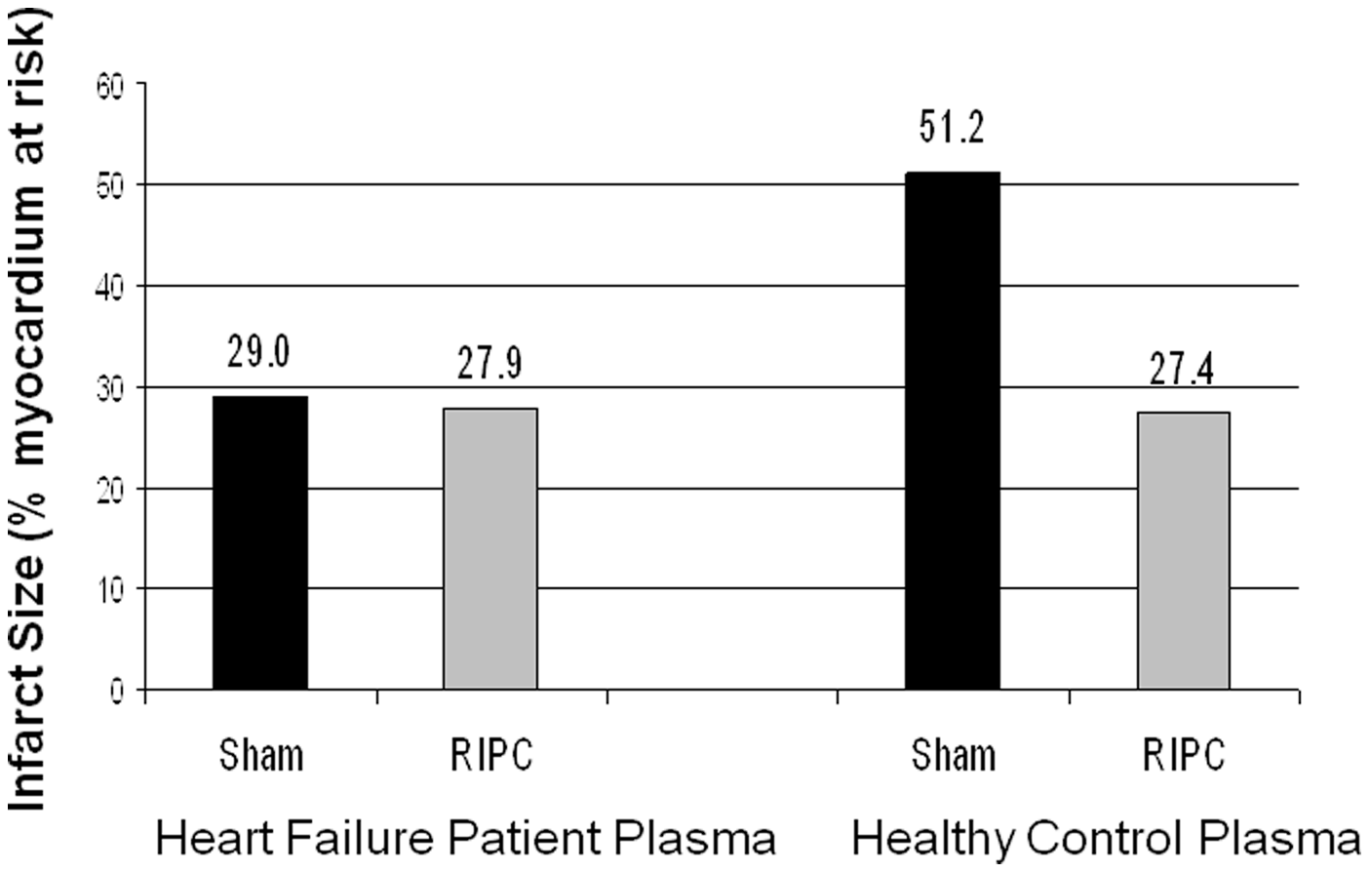
Langendorff mouse heart infarct size after perfusion with dialysate from heart failure patients versus healthy controls. Heart failure patient dialysate, irrespective of RIPC or sham treatment, reduced infarct to the same extent as the dialysate from RIPC-treated healthy controls, as compared to sham treated healthy controls.

Using this Langendorff model, we undertook a further exploratory comparison of infarct size following perfusion with dialysate derived from our heart failure study population versus infarct size following perfusion with dialysate derived from plasma from healthy controls undergoing sham/RIPC intervention. The infarct size observed in hearts perfused with dialysate from our heart failure patients was nearly 45% less than the infarct size observed in hearts perfused with healthy control dialysate following sham intervention. Moreover, the infarct area seen in our study approximates the infarct area seen with exposure to dialysate from healthy control subjects following RIPC intervention (Figure 3).

**Figure 4:**
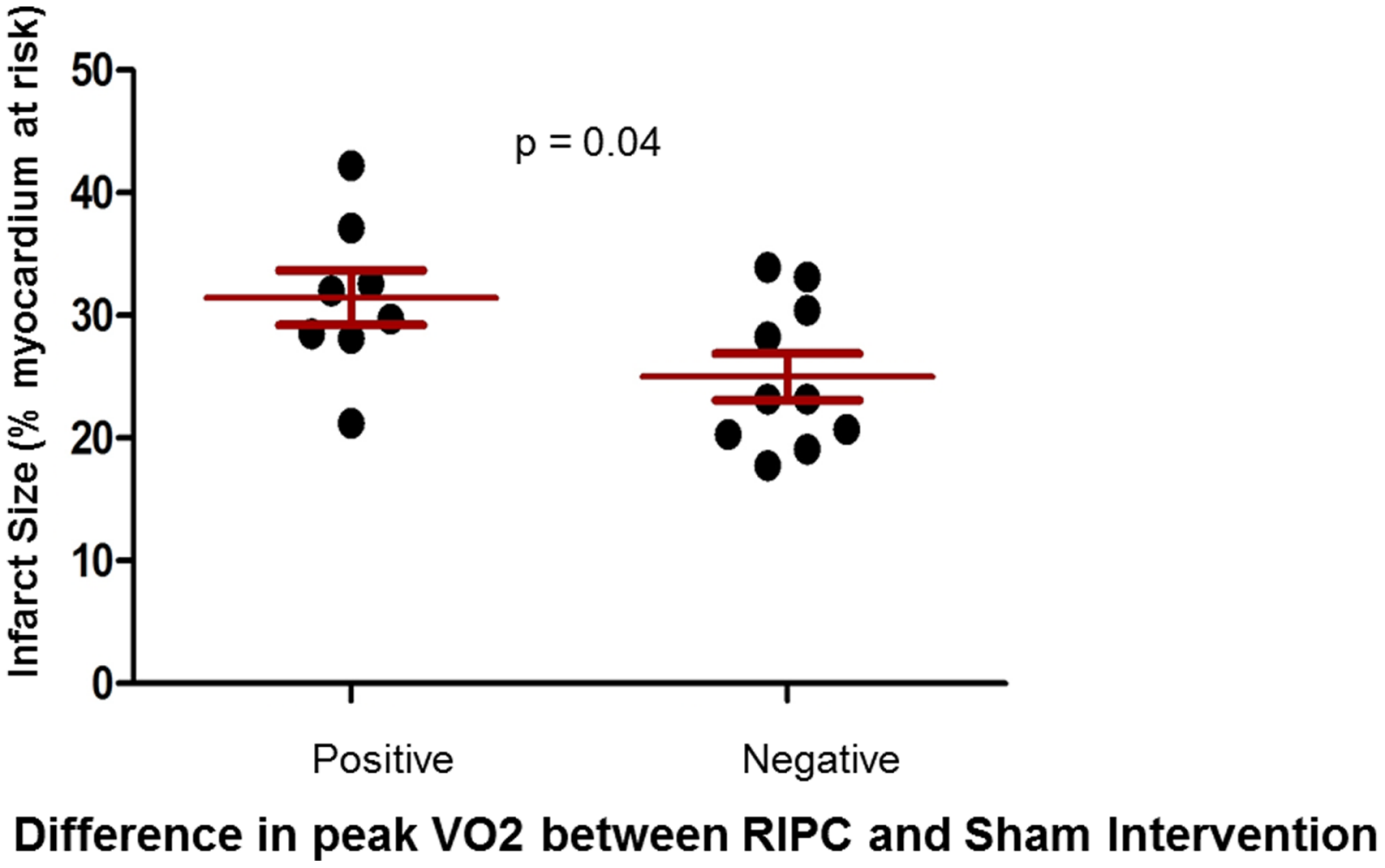
Infarct size stratified by the effect of RIPC on exercise performance. In the Langendorff model, mean infarct size was significantly smaller after perfusion with dialysate from the subgroup of patients who had no improvement in exercise performance following RIPC treatment. Data are presented as mean +/− SD % of infarcted myocardium.

In order to determine whether the apparent lack of effect of RIPC in our study population could be explained by a high degree of baseline preconditioning, we assessed infarct size in the Langendorff model according to clinical response to RIPC treatment. In this post hoc analysis, mouse infarct size was significantly smaller among the subgroup of patients who had no improvement in exercise capacity following RIPC intervention, suggesting that the overall absence of a clinical response to RIPC in our study population may relate to a higher degree of preconditioning at baseline for the majority of patients (Figure 4).

## Discussion

In this randomized crossover pilot study, RIPC was not associated with improvements in objective measures of exercise capacity in ambulatory HF patients with left ventricular dysfunction. Specifically, there was no overall benefit with respect to any of the prognostically important exercise variables, including peak VO2, exercise duration, workload achieved or VE/VCO2 slope during cardiopulmonary stress testing. A principle finding from our Langendorff sub-study was that RIPC in this study population does not appear to confer additional protection against ischemia reperfusion injury through release of circulating preconditioning factor(s). This observation is in contrast with the effects of RIPC in a healthy control population [23]; moreover our results suggest that patients with chronic HF may already be relatively preconditioned.

To our knowledge, this is the first clinical trial to evaluate the effect of RIPC in a heart failure population. There is a growing body of evidence that suggests RIPC induced by transient limb ischemia, similar to the protocol used in our study, is associated with a significant reduction in end organ injury due to a variety of ischemic stressors. One of the earliest clinical studies of RIPC was a randomized controlled trial of 37 pediatric patients undergoing cardiopulmonary bypass for corrective cardiac surgery [17]. In this study, four 5-minute cycles of limb ischemia and reperfusion resulted in less troponin elevation, lower inotrope requirements, and better lung function in the early post-operative setting. RIPC with lower limb ischemia was also shown to attenuate myocardial and renal injury in a randomized controlled trial of 82 patients undergoing elective abdominal aortic aneurysm repair [19]. These early trials have supported the observation that RIPC confers multisystem protection in an ischemic environment.

Maximal exercise performance is limited by ‘relative’ ischemia of skeletal muscle and is associated with tissue hypoxia and lactic acid accumulation, and thus can be considered a form of ischemic stress. Recently, the potential benefits of ischemic preconditioning have extended into the domain of exercise performance in healthy individuals. de Groot and colleagues showed that ischemic preconditioning of each leg prior to bicycle exercise testing improved both maximal workload and maximal oxygen consumption [22] in healthy volunteers. Members of our group have shown that RIPC induced by transient upper limb ischemia immediately prior to exercise was associated with significant improvements in competitive swim times in elite athletes [23]. Furthermore, dialyzed serum taken from the swimmers following RIPC (but not serum taken before) was shown to carry a circulating “protective factor” that significantly reduced infarct size in a mouse Langendorff model of ischemia-reperfusion [23], confirming the importance of humoral-mediated pathways discussed earlier [14]–[16]. Prior to performing our study, we hypothesized that RIPC may have a favorable impact on exercise capacity of HF patients who are subject to musculoskeletal and cardiac ischemia as they reach an anaerobic state at peak exercise. These patients have poor exercise tolerance secondary to reduced cardiac output, abnormalities in myocyte fibre composition, and reduced mitochondrial density [7]–[9]. Any intervention to improve exercise performance in a HF population with various physiologic limitations could potentially translate into important clinical benefits.

Why then was there no overall impact of RIPC on exercise capacity in this study, despite using a conventional preconditioning protocol? The answer to this question may be multifactorial, but our Langendorff study provides important new evidence to suggest that these patients may already be in a ‘preconditioned state’. Indeed, our results show that plasma obtained after sham intervention already contained dialyzable circulating cardioprotective factors characteristic of those liberated by remote preconditioning stimuli [14]–[16]. Consequently, overall the level of cardioprotection observed in control dialysate from the heart failure patients was similar to that seen after RIPC in normal volunteers, and was unaffected by additional RIPC. The reason for this is less clear however. There is emerging evidence that vigorous exercise in healthy subjects itself is a stimulus for release of cardioprotective factors [24]. We speculate that daily activities in HF patients may act as a similar stimulus to the release of cardioprotective factors as a result of skeletal muscle ischemia. It is also possible that the heart may act as a paracrine organ for the release of cardioprotective factors. For example, Dickson and colleagues have shown that coronary effluent from hearts subjected to brief periods of ischemia is cardioprotective in an untreated acceptor heart [25].

Irrespective of where the circulating factors originate, our data do suggest that there may be a subgroup of heart failure patients that might respond to RIPC. Although the numbers were small, when we compared those that had significantly improved exercise performance with RIPC, versus those that did not, there was also a significant difference in the level of cardioprotection afforded by their plasma dialysate in the mouse Langendorff studies. Plasma from patients that showed improvement in exercise capacity had lower baseline protection against ischemia, suggesting that their degree of baseline preconditioning was lower, and hence their capacity to respond to RIPC was higher.

It is also important to note, that while we were able to demonstrate the presence of circulating cardioprotective factors in vitro, we cannot confirm that they are the same as those induced by RIPC, nor can we confirm that they have any effect in-vivo in our HF patients. In pre-clinical studies, older age and underlying cardiac dysfunction have been shown to result in abnormal mitochondrial permeability transition pore and kATP channel function that may blunt the effects of RIPC [26], [27]. There are also well documented ultrastructural changes in peripheral skeletal muscle associated with impairments in oxidative capacity [7]–[9], [28], and the implications for ischemic preconditioning remain unclear. Failing myocardium also undergoes ultrastructural adaptation characterized by fetal gene reprogramming, with a decreased rate of aerobic metabolism and relative resistance to ischemia [29], [30]. Coupled with the observation that mitochondrial kATP channels are endogenously activated in heart failure [27], all of this data suggest that in the clinical domain, RIPC may not yield significant additional protection from ischemic stress in a relatively ‘preconditioned’ HF state. While our pilot data show that there appears to be a subgroup of patients that benefit most from RIPC (those with low baseline preconditioning), in the absence of a clinical test of ‘pre-existing preconditioning’ the widespread adoption of RIPC as a clinical tool will require demonstration of effect in large, unselected, cohort studies. In this regard, it is likely that our study was underpowered to detect a small, but still meaningful difference in peak VO2 between RIPC intervention and sham control exercise tests. Based on the results from our pilot data, future trials would need an approximately 4–5 fold our sample size to have at least 80% power to detect a clinically important difference of 1–2 mL/kg/min in peak VO2. Smaller degrees of improvement would not likely translate into better transplant free survival [12].

Our study has a number of important limitations that may warrant caution in the interpretation of our results. Our post-hoc subgroup analysis of ‘responders’ versus ‘non-responders’ to RIPC, is not only somewhat artificial in terms of examining responses in a binary fashion, but also does not allow meaningful analysis of factors associated with those response, beyond the reported differences in cardioprotective activity of their plasma. A number of comorbidities and medications have been associated with either blunted or enhanced responses to ischemia-reperfusion injury and preconditioning [31], [32]. As we did not exclude patients with comorbidities such as hypertension, dyslipidemia or left ventricular hypertrophy, and we did not perform a medication washout prior to testing, it is possible that our results may have been confounded. The impact of residual medications such as ACE inhibitors and statins in the Langendorff perfusate is indeterminate, and it is conceivable that these therapies may have modified the preconditioning response in the mouse heart. Although our model has previously been used to demonstrate the presence of a humoral mediator of RIPC [23], it is also possible that some of the larger sized key mediators of preconditioning were filtered from plasma while preparing the dialysate. For example, Giricz and colleagues recently demonstrated that extracellular vesicles (exosomes and microvesicles) may be necessary to confer protection in a rat heart Langendorff model of ischemic preconditioning [33]. Furthermore, we did not measure lactic acid levels to confirm a state of relative hypoxia with anaerobic metabolism at peak exercise. However, our cardiopulmonary exercise protocol with respiratory gas analysis did allow for an estimation of anaerobic threshold as a surrogate for lactic acidosis. Finally, patients in our trial had considerable variability in their inter-test intervals. Although we attempted to repeat paired exercise tests within 4 weeks from enrollment, we were unable to achieve this for all patients, providing the opportunity for bias due to changes in treatment or clinical status between tests.

In summary, we conducted the first randomized controlled trial of RIPC in ambulatory patients with HF and left ventricular systolic dysfunction. In this study, RIPC was not associated with improvements in objective measures of exercise capacity. The apparent absence of an effect of RIPC may relate to a high degree of baseline preconditioning in our patient population, and those with lesser degrees may be benefit with RIPC. However large, adequately powered clinical trials and further mechanistic translational studies are needed before the effects of RIPC in HF can be established definitively.

## Supporting Information

Checklist S1 **CONSORT Checklist relevant to the main study protocol.**
(DOC)

Protocol S1 **Study rationale and summary of the study and sub-study protocols.**
(DOC)
