# Supplementary material for: A Randomized Pilot Trial of Remote Ischemic Preconditioning in Heart Failure with Reduced Ejection Fraction
Source: PLoS One. 2014 Sep 2;9(9):e105361. doi: 10.1371/journal.pone.0105361 (PMC4152122; doi:10.1371/journal.pone.0105361)
Supplement: Protocol S1 — Study rationale and summary of the study and sub-study protocols. (DOC) [file pone.0105361.s002.doc]

Effect of remote ischemic preconditioning on exercise capacity in patients with heart failure: a double blind randomized control trial

Background and Rationale:

# Remote Ischemic Preconditioning

Remote ischemic preconditioning (RIPC) is the process in which a transient, sub-lethal reduction in blood flow and oxygen delivery to tissues in one area of the body offers protection to other remote tissues from a subsequent episode of sustained ischemia. For example, animal studies have convincingly demonstrated that periodic peripheral limb ischemia, typically four cycles of 5 minutes each, followed by 5 minutes of reperfusion, reduces myocardial injury following cardiopulmonary bypass and decreases ischemia-reperfusion injury in the setting of myocardial infarction. Human studies in children and adults undergoing elective cardiac surgery have shown that three to four x 5-minute cycles of limb ischemia induced by blood pressure cuff inflation results in significantly less post-operative myocardial injury. In another recent study of adults undergoing elective percutaneous coronary intervention, RIPC was associated with less pain, less myocardial damage, and decreased major adverse cardiovascular events at 6 months. RIPC with this technique has been used in a variety of other clinical experiments with no known adverse effects, and therefore remains an attractive, non-invasive intervention with significant therapeutic potential. While the precise mechanisms are not known, available evidence suggests that factors are released into circulation during the preconditioning stimulus that exert their effects via intracellular kinases and alterations in mitochondrial function.

Expanding the concept that RIPC acts as a modulator of end-organ metabolism, the effects of ischemic preconditioning were recently tested in a model of exercise induced arterial hypoxemia in elite swimmers. Because swimmers must hold their breath during different phases their stroke cycle, their ventilation becomes limited in relation to metabolic demand, resulting in appreciable decreases in the arterial partial pressure of oxygen. It was therefore hypothesized that the effects of exercise induced arterial hypoxemia may be ameliorated, in part, by ischemic preconditioning. Notably, in a randomized controlled trial, four cycles of blood pressure cuff-induced ischemia for 5 minutes each resulted in significantly improved competitive swim times compared to sham controls in these athletes. This suggests that RIPC may provide a stimulus that helps buffer cardiac, respiratory and peripheral skeletal muscles under hypoxic conditions. Conceptually then, ischemic preconditioning may have salutary effects in other populations, including patients with heart failure for whom exercise limitations have important prognostic implications.

# Exercise Capacity in Heart Failure

The study described in detail below will examine the possible effects of RIPC in patients with left ventricular systolic dysfunction and symptomatic heart failure. Despite advances in pharmacologic and device-based therapies, patients with heart failure continue to be burdened with exertional fatigue, dyspnea and exercise intolerance. These symptoms, along with objective findings of diminished exercise capacity are unequivocally associated with decreased quality of life and worsened survival. Peak oxygen consumption (peak VO2) and measures of ventilatory efficiency during exercise remain powerful predictors of mortality or need for cardiac transplantation and/or mechanical circulatory support.

Exercise intolerance in heart failure patients is thought to be multi-factorial. Assuming normal pulmonary gas exchange, VO2 is a function of cardiac output and skeletal muscle oxygen uptake and metabolism, both of which are impaired in the setting of heart failure. During exercise, the inability to adequately increase cardiac output and skeletal muscle blood flow is associated with early anaerobic metabolism, lactic acid production and muscle fatigue, commensurate with restricted peak VO2. Compounding this circulatory dysfunction, patients with heart failure typically develop adverse changes in skeletal muscle physiology. For example, there is generalized muscle atrophy over time, a decreased proportion of fatigue-resistant type I muscle fibers, and alterations of mitochondrial function leading to inefficient use of high-energy phosphates. As such, the alterations in peripheral muscle structure and function result in impaired VO2, even if cardiac output is increased pharmacologically with the administration of inotropes or vasodilators.

Aerobic fitness training as a means of conditioning the cardiovascular system and improving skeletal muscle function has been shown to improve quality of life, exercise duration, VO2, and other indices of exercise performance. More recently, in the HF Action study, a 2300 patient randomized controlled trial of exercise training versus usual care in patients with heart failure and reduced ejection fraction, exercise training was associated with a 10% reduction in mortality or cardiovascular hospitalization after adjusting for other prognostic variables. If ischemic preconditioning as a therapeutic intervention was found to improve exercise capacity in the heart failure population, it could translate into significant clinical benefits. RIPC has not previously been studied in patients with heart failure, and therefore merits evaluation.

**Research Purpose**

The main purpose of this pilot study is to determine whether RIPC affects measures of exercise capacity in patients with non-ischemic left ventricular dysfunction and symptomatic heart failure. Differential effects on various exercise performance measures could also provide mechanistic insights into the benefits seen with RIPC.

A secondary aim of the study is to determine whether the protective humoral factor, known to be released into the bloodstream of healthy individuals during RIPC, is similarly released by patients with heart failure. This will require a sub-study of the effect of dialysed patient plasma on explanted mice hearts in a Langendorff preparation. Any protective effects of RIPC in our Langendorff heart model will be correlated with exercise performance in heart failure patients.

If we find that RIPC is associated with improvements in cardiac and skeletal muscle function and exercise capacity in this study, we plan on conducting a larger randomized controlled trial to assess the therapeutic effects RIPC on clinical outcomes in a broader spectrum of heart failure patients.

Study participants would include patients followed in the Heart Function Clinic at the Toronto General Hospital, a referral center for heart failure management and cardiac transplantation evaluation. This multidisciplinary clinic actively follows approximately 850 patients and sees 25-30 new referrals per month. Clinic patients undergo routine assessment, including cardiopulmonary exercise testing by a trained exercise physiologist at regular intervals as dictated by clinical circumstances. In general, the target population has a baseline risk of death in excess of 30% per year and would be expected to have at least a moderate impairment in exercise capacity. Consequently, Heart Function Clinic patients represent an ideal sample population in which to study the effects of RIPC on exercise performance.

## Hypothesis

1. We hypothesize that RIPC induced by transient upper limb ischemia will improve exercise performance measures in patients with non-ischemic cardiomyopathy and symptomatic heart failure.
2. Langendorf mouse heart preparations perfused with dialysed plasma from patients after RIPC treatment will be more resistant to ischemic injury than mouse heart preparations perfused with dialysed plasma obtained after ‘sham’ RIPC.

**Methods**

*Study Design*

This pilot study will be a randomized double blind cross-over trial of RIPC in ambulatory heart failure patients undergoing cardiopulmonary stress testing. One-half of study patients will be randomly assigned to RIPC treatment in the first session, and the second half of the patients will receive sham preconditioning prior to exercise testing. The following week, patients will undergo whichever protocol they had not completed in the first session, prior to repeat exercise testing. In this manner, patients will be exposed to both the RIPC and sham treatments.

Investigators and patients will be blinded to treatment allocation. The specific RIPC intervention will include four 5-minute cycles of upper limb blood pressure cuff inflation to 20mmHg above systolic blood pressure to induce forearm ischemia. Each 5-minute cycle of ischemia will be followed by 5 minutes of reperfusion (blood pressure cuff fully deflated). The sham-control arm similarly will include four 5-minute cycles of blood pressure cuff inflation to 10mmHg only, interspersed with 5 minutes of cuff deflation. Following blood sampling (see below), the, patients will then perform a routine cardiopulmonary exercise test under the supervision of a single dedicated exercise physiologist who will be blinded to treatment assignment.

The cardiopulmonary exercise study consists of a standard stationary bicycle ramp protocol in which workload increases by 10 watts per minute. The study is symptom- limited once a target respiratory exchange ratio (RER) of ≥ 1.05 is achieved. The RER ≥ 1.05 indicates that the patient has exercised with sufficient intensity to induce lactic acidosis as a byproduct of anaerobic metabolism. The exercise study would also terminated prematurely for any of the following: hypotensive response (≥ 10mmHg drop in systolic blood pressure) to increasing work load, lightheadedness, signs of poor perfusion, signs of significant ischemia on the ECG (≥ 2mm horizontal/downsloping ST segment depression or ≥ 1mm ST segment elevation in non-infarct leads), significant brady or tachyarrhythmia including ventricular tachycardia, hypertensive response to exercise (systolic blood pressure ≥250 mmHg or diastolic blood pressure ≥ 110mmHg) or upon patient request.

# Study Patients

Ambulatory patients from the Toronto General Hospital Heart Function Clinic scheduled for routine cardiopulmonary stress test will be approached and offered participation. Appropriate patients will meet the following inclusion and exclusion criteria:

Inclusion criteria:

Male and females >18yrs of age

Non-ischemic cardiomyopathy

LV ejection fraction <40%

New York Heart Association functional class II-IV

Clinically stable

Exclusion criteria:

Recent cardiovascular hospitalization within the previous 4 weeks

New heart failure diagnosis within previous 12 weeks

Ischemic cause for cardiomyopathy or active ischemia

Diabetes mellitus

Peripheral neuropathy

Not able to ambulate or complete exercise bicycle protocol for non-cardiovascular reasons

Presence of a ventricular assist device

Uncontrolled brady or tachyarrythmias

Uncontrolled hypertension (systolic blood pressure ≥ 200mmHg)

Severe obstructive valve lesion

Significant LV outflow tract gradient

Aortic dissection

Acute myocarditis/pericarditis

Baseline clinical characteristics will be collected, including age, gender, heart failure duration and etiology, last available LVEF, NYHA functional class, history of hypertension, atrial fibrillation/flutter, pacemaker/biventricular pacemaker, implantable defibrillator, non-cardiac comorbidities, medications, weight, height, blood pressure, heart rate, serum creatinine, sodium, BNP.

# Study Endpoints

Clinical endpoints will be ascertained at the conclusion of the cardiopulmonary stress test. The primary endpoint will be peak VO2 in patients achieving a respiratory exchange ratio >1.05. Secondary endpoints include other measures of exercise performance: ventilatory threshold, peak VE/VCO2 slope, workload achieved in Watts, total exercise time and oxygen uptake efficiency slope. We will also assess serum lactate post stress to directly determine the effect of RIPC on lactic acid production under anaerobic conditions.

# Sample Size and Data Analysis

This pilot study will be conducted with a sample size of 44 patients to determine baseline exercise parameter and variance values in our study population. Assuming that our patients are similar to contemporary heart failure patients evaluated in exercise training trials, mean baseline exercise peak VO2 is expected to be approximately 14.0 –15.0 mL/kg/min, with an estimated standard deviation of 2.5 mL/kg/min. A 44 patient cross-over trial would therefore provide 80% power to detect a difference of 1.5 mL/kg/min, which represents a clinically significant ~10% change in peak VO2 (2-tailed alpha level 0.05).

Continuous variables will be summarized using means and standard deviations and contiguous variables will be summarized as proportions. Student’s T test and chi squares will be used to determine differences between continuous and categorical variables, respectively. Pre-specified subgroup analyses will assess the effect of RIPC on patients with moderately severe exercise limitation (sham peak VO2 <14mL/kg/min) or very severe exercise limitation (sham peak VO2 <10mL/kg/min), as well as its effect on patients taking beta blockers versus those not taking beta blockers.

# Sub-study Design

*i) Preparation of plasma dialysate:*

25 of blood will be taken after the RIPC or sham RIPC protocol has been completed. Each sample will be anonymised and coded according to treatment assignment, stored on ice and transferred to the Redington laboratory in the MacMaster building, Sickkids. Subsequently, the plasma fraction will be separated by centrifugation (3,000 *g* for 20 min). To prepare dialysate, 12-15 of plasma will be placed in dialysis tubing with a 12-14 kDa cut-off membrane (Spectrapor, USA), dialysed against a 20 fold volume of Krebs buffer (240-300 mL) while stirred overnight at 4^o^C, and stored at -80^o^C**.** Prior to perfusion of the mouse hearts, D-glucose, NaHCO3 and EDTA are added to a final concentration of 15mM, 25 mM and 0.5mM, respectively to the dialysate.

*ii) Mouse langendorff:*

After a 20-min stabilization period, isolated hearts will be perfused for 30 min using dialysate, then washed out for 5min, and then subjected to 30 min of no-flow global ischemia followed by 60min of reperfusion. HHeHemodynamic measurements, including heart rate (HR), peak left ventricular pressure (LVP), maximum rate of contraction (+dP/dtmax), maximum rate of relaxation (-dP/dtmin), and LVEDP will be recorded on a data acquisition system (PowerLab, ADInstruments) throughout the procedure. Coronary flow rate will be calculated by measuring the amount of effluent emerging from the heart per minute (ml/min). Myocardial infarction size will be measured in heart slices stained with 1.25% triphenyltetrazolium chloride (TTC) in 200mM Tris pH 7.4 for 15 min in 37^o^C water bath. After fixation for 4-6 hours in 10% neutral buffered formaldehyde, both sides of each slice will photographed by electronic scanning (CanoScan 4400F) and the infarct area measured by automated planimetry using Adobe Photoshop® CS2 software. All personnel involved in the preparation and assessment will be blinded to treatment assignment.

# **Specific Patient Risks**

# Exercise Testing

Patients with advanced heart failure routinely undergo cardiopulmonary stress testing for diagnostic and prognostic purposes. Exercise testing is considered safe in the broad spectrum of patients being evaluated for cardiac disease, with a risk of death or non-fatal myocardial infarction estimated at 1/2500. Patients are electrocardiographically monitored, and symptoms, heart rate and blood pressure are assessed at regular intervals throughout the test.

In the heart failure population, a large proportion of patients have implantable cardioverter defibrillators (ICDs). There is a theoretical risk of receiving a shock during exercise, however ICDs are not considered a contraindication to stress testing provided patients are supervised, and that exercise heart rate does not exceed the arrhythmia detection threshold rate. In our practice, patients with ICDs are carefully supervised to ensure they do not approach their set arrhythmia detection rate, with a typical margin of safety of 10 beats per minute.

Ischemic preconditioning with blood pressure cuff induced skeletal muscle ischemia may result in brief local discomfort, a tingling sensation or transient muscle weakness in the affected arm. There are no known longer-term risks associated with this intervention.

# Blood sampling

Blood draws for laboratory measurements may be associated with discomfort and/or bruising and the possibility of infection. To minimize risks, puncture sites will be disinfected with alcohol swabs and a band-aid applied for hemostasis. All materials used in blood punctures will be used once and discarded into a biohazard waste container.

# **Anonymity and Confidentiality**

Participating patients will be assigned a unique identifying code to preserve anonymity. Data will be collected and maintained by the investigators only, and all paper and electronic records will be kept in a secure location at the Toronto General Hospital.

# **Compensation**

Patients will be reimbursed for parking costs but will receive no other compensation for their participation in this study.

# **Informed Consent**

Information will be provided to participants in a manner that is understandable to them. All aspects of the project including rationale and procedures involved will be carefully explained. Patients will understand that their participation in this study is completely voluntary and that they are free to withdraw at any time without repercussion. A decision not to participate will in no way affect their care through the Heart Function Clinic. Participating patients will receive a copy of the letter explaining the study as well as the signed consent form.
